# Gaze Coordination of Groups in Dynamic Events – A Tool to Facilitate Analyses of Simultaneous Gazes Within a Team

**DOI:** 10.3389/fpsyg.2021.656388

**Published:** 2021-03-17

**Authors:** Frowin Fasold, André Nicklas, Florian Seifriz, Karsten Schul, Benjamin Noël, Paula Aschendorf, Stefanie Klatt

**Affiliations:** ^1^Institute of Exercise Training and Sport Informatics, German Sport University Cologne, Cologne, Germany; ^2^Institute of Sports Science, University of Rostock, Rostock, Germany

**Keywords:** team sports, officials, gaze behavior, teamwork, eye-tracking

## Abstract

The performance and the success of a group working as a team on a common goal depends on the individuals’ skills and the collective coordination of their abilities. On a perceptual level, individual gaze behavior is reasonably well investigated. However, the coordination of visual skills in a team has been investigated only in laboratory studies and the practical examination and knowledge transfer to field studies or the applicability in real-life situations have so far been neglected. This is mainly due to the fact that a methodological approach along with a suitable evaluation procedure to analyze the gaze coordination within a team in highly dynamic events outside the lab, is still missing. Thus, this study was conducted to develop a tool to investigate the coordinated gaze behavior within a team of three human beings acting with a common goal in a dynamic real-world scenario. This team was a (three-person) basketball referee team adjudicating a game. Using mobile eye-tracking devices and an indigenously designed software tool for the simultaneous analysis of the gaze data of three participants, allowed, for the first time, the simultaneous investigation of the coordinated gaze behavior of three people in a highly dynamic setting. Overall, the study provides a new and innovative method to investigate the coordinated gaze behavior of a three-person team in specific tasks. This method is also applicable to investigate research questions about teams in dynamic real-world scenarios and get a deeper look at interactions and behavior patterns of human beings in group settings (for example, in team sports).

## Introduction

If a group of human beings tries to fulfill the requirements of a specific task, e.g., to solve a problem, or to reach a specific performance criterion, the members of this group can coordinate their abilities and skills to optimize their behavior and/or performance. For instance, lifeguards coordinating scanning of water surfaces to minimize the risk of a failure to perceive a drowning person, or in a dangerous situation during rush hour traffic on the road, the role of co-driver in observing the traffic to minimize the risk of a road accident. Similarly, in team sports, coordination of visual skills could be relevant for the overall performance of both, a team of sportspeople and/or referees.

The coordination of gaze among group members or within a team has almost exclusively been studied only in different experimental laboratory investigations. In a police-training simulation (on computer screens), for example, Neider et al. (2010) showed that different options for sharing information (verbal communication, shared gaze *via* gaze cursors of partners fixations), can influence performance in spatial referencing. The authors stated that verbal and visual information can be helpful for interpersonal coordination. An earlier study showed that in simple perceptual search tasks, the coordination of gazes through the observation of a partner’s gaze leads to superior performances compared to individual search (Brennan et al., 2008). Bahrami et al. (2010) demonstrated in a low-level perceptual task (visual detection of a target stimulus) that team (dyads, i.e., two individuals) decisions are better than the decisions made by just one observer. A necessary condition for better performance is the opportunity of free communication within the dyads.

Other than the aforementioned studies, Macdonald and Tatler (2018) provided a description of the non-verbal communication cues that people use in real-world situations. They investigated the synchronized gaze behavior of a pair engaged in the activity of making a cake and observed how the roles of the subjects (chef and gatherer) influence the gaze behavior in this (social) interaction task (Macdonald and Tatler, 2018). Other than this study, almost no research concerning the interaction of gazes or the gaze coordination of people working in a team setting has been published so far. In particular, there has been no application-oriented method which is presented to analyze teams’ gaze behavior, even though, the meta-analyses by Mann et al. (2007) as well as by Gegenfurtner et al. (2011) has suggested that specific cognitive abilities are better investigated in natural environments. Risko et al. (2016) additionally noted the absence of applicability of laboratory studies to real-world environments in the area of social attention.

Over the last few decades, technological advances in mobile eye tracking systems have made it easier to conduct field studies on gaze behavior [Kredel et al., 2017, also see Wan et al. (2019)]. In recent times, software solutions for (automated) data analyses have been widely available (e.g., Panetta et al., 2019, 2020). However, there is almost no procedure to evaluate the synchronized tracking of gaze behavior within teams (through eye tracking) in more or less dynamic situations. This shows that although the analysis of individual gaze behavior has been facilitated, it has not led to an increase of research on gaze behavior within groups.

Interestingly, this is also the case in research in areas like sports science and sports psychology, where eye tracking has become an often-used method to analyze athletes’ or referees’ gaze behavior. Recent reviews about gaze behavior in sports (Kredel et al., 2017; Hüttermann et al., 2018) have given an extensive overview on contents, methods, and practical applications which have been used in the past decades. Notably, all the studies included in the reviews, focused on individual parameters and joint activities of groups or team members. Considering that the performance in team sports (athletes and officials/referees) is primarily based on the coordination and interaction of individual skills performed by each team member, the lack of research on the subject is surprising.

However, there has been one study which investigated the coordinated gaze behavior of handball referees adjudicating a game (Fasold et al., 2018). While Fasold et al. (2018) showed that the analysis of the coordinated gaze of a dyad can be done well by using available software tools (e.g., eye-tracking device application, KINOVEA), it was evident that the observation of more than one person at the same time requires a well-structured experimental set-up. Furthermore, this procedure is associated with extensive processing of high volumes of data, especially considering that analyzing data of gaze behavior in highly dynamic situations is mainly done manually as a frame-by-frame analysis. Manual analysis is possibly the main reason for the research gap with regard to the analysis of the gaze behavior of two or more individuals in a natural environment. This is also related to the fact that a method to technically analyze the gazes of more than two people in a time-effective way is missing [considering that freeware solutions, most often, only allow the analysis of a dyad, see Fasold et al. (2018)].

The current study expands the approach of Fasold et al. (2018) by analyzing the synchronized gaze behavior of three individuals working together in a team. In contrast to lab-based studies, which allow high-frequency data collection and algorithmic based data analyses (Mele and Federici, 2012), in field studies it is often not possible to use automated analyses due to the dynamic movements, the accelerations, and the three-dimensional and ever changing areas of interest (AOI). While previous approaches for automated analyses of individual gaze behaviors in more or less dynamic environments do exist (e.g., Panetta et al., 2019), a manual frame-by-frame analysis is necessary to investigate the simultaneous gaze behavior of three referees. This kind of analysis, although time-consuming, is reliable and functional for sports practice (cf. Fasold et al., 2018). Therefore, the main focus of the current study was on the development of a reasonable way of analyzing data and figuring out a way to handle the large dataset (three eye-tracking devices).

### Study

**FIGURE 1:**
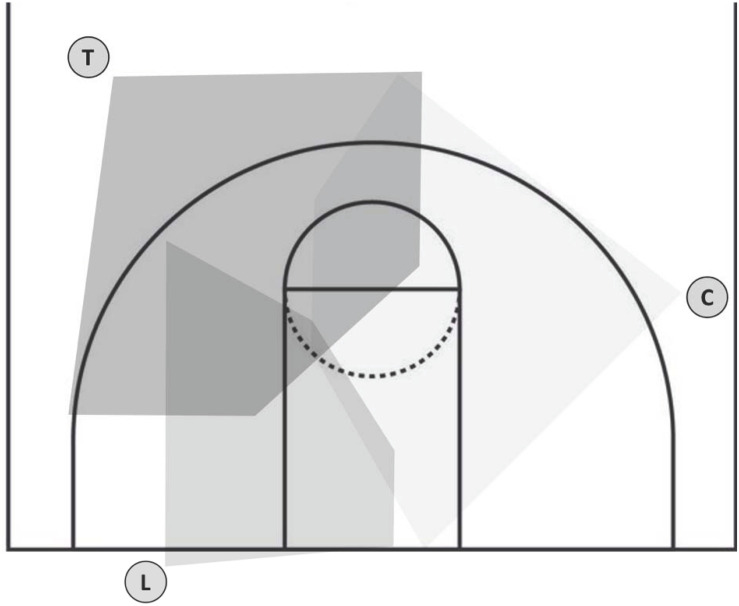
Coverage of gaze areas of the three referees’ positions (L, lead; T, trail; C, center) in the 3PO system, predefined by the guidelines for referees [modified figure according to FIBA (2016), p. 178)].

In investigating performances in team sports, usually, it is mainly the athletes who are the primary focus. But referees, who play a vital role in successfully conducting a game, have to deliver high performances in judging a large number of interactions in highly dynamic situations involving a lot of cues/athletes (e.g., Plessner and MacMahon, 2013). The relevance of the visual system in conducting refereeing tasks is well known, as documented by MacMahon et al.(2015, p. 47): “Perceiving, and in most cases seeing, is at the root of any judgment and decision in refereeing in sports.” The assumption that groups of people can perceive more than an individual would, is derived from the refereeing regulations of various sports. The regulations of a multitude of (professional) sports demand the presence of a team of referees, rather than an individual referee (e.g., two referees in volleyball or team handball, three referees in basketball or ice hockey). Thus, for our study, we chose to simultaneously observe the gaze behavior of three basketball referees by means of three individual mobile eye-tracking systems for each of them during a preparation game. In professional basketball, usually the 3-Person Officiating system (3PO) is used, meaning three referees are systematically assigned to the court in order to avoid as little action as possible (FIBA, 2016). Almost always, it’s the FIBA which defines the zone/area which has to be covered by a specific referee (see Figure 1), and thus, it also determines how the referees should coordinate their gaze behavior (FIBA, 2016).

Despite these clear guidelines, it is actually unclear if a team of referees is able to simultaneously cover the court in the most optimum manner. To test the applicability of a new method to analyze the simultaneous gaze behavior of three people, we analyzed the referees’ gaze behavior and compared it to the FIBA guidelines for refereeing. It is noteworthy that FIBA (2016) clarifies that the ground coverage by referees in an active game is not static and has to be adapted according to the dynamic flow of the game. Overlapping and/or dual coverage of some areas is not necessarily wrong and may be indicative of the functioning of the way that the referee team works.

## Methods

This study is the starting point of a larger project which evaluates the on-court visual behavior and social interaction among the referees in the team. The simultaneous coordination of gazes is one part of this application-oriented investigation, the other being the large amount of data resulting from such interaction. So far, a method to observe this interaction or measure the data has not been developed. Therefore, we developed a tool to facilitate a frame-by-frame analyses of simultaneous gaze behavior of several observers and tested it on a representative data set.

### Tool Development

In order to analyze the simultaneous video streams (gaze data) of two people working as a team in a natural environment, a software named KINOVEA was applied in the study by Fasold et al. (2018). However, that method only allows the simultaneous analysis of two video streams. Moreover, while analyzing data, frame-by-frame video players’ analysis is not sufficient because it only allows watching recordings of the several gazes frame-by-frame, without any options to save the results of such analyses. That is, all data must be separately entered and saved by means of an additional computer program. Such a procedure–switching from one video stream to another or watching several gaze recordings of the same situation in succession and switching from one computer program to another in order to enter data–is time-consuming and prone to errors.

Initially for the current project, but with a practical applicability beyond that for many future eye-tracking projects, we developed a tool enabling the simultaneous analysis of four video streams. This tool allows observation of three video streams of gaze data recorded with the eye-tracking devices (Referees A, B, and C) and a fourth stream showing the complete scene video, in the current case, recorded from the stand in the middle of the playing field (just for the validation of the game situations). This tool offers the possibility to play the videos frame by frame with the inserted gaze points extracted from the eye-tracking software (Pupil Player). We developed the tool using components of Delphi XE3, PasLibVlc^1^ and the commercial TMS Component Studio^2^. The complete source code is available in a public GIT repository^3^. From the TMS Component Studio, we used only the components of the feature-rich user interface to create a modern user interface for our tool. Some features of the PasLibVlc are also used in the well-known VLC-Player. This ensures that for the purpose of our tool, most video formats can be played back seamlessly and this can also be used with other eye-tracking systems.

**FIGURE 2:**
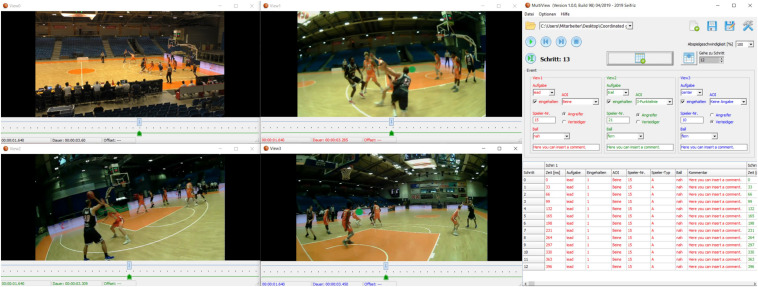
Screenshot of the user interface of the analysis tool enabling the analysis of four video streams at the same time.

Our main goal for the software-user interface was to create a simple and efficient collection of the relevant data from various observers. Therefore, we developed a project structure to save all the necessary data of the used video files in one repository. The project properties included, among other things, the video file location, the position of each window of the video streams, and the synchronized start frame of the video streams. These properties were saved in a separate project file. After saving the files, an analysis could be started again after an interruption without much effort by simply loading the project property file. In order to avoid any data loss, the current status of the analysis was saved after editing each frame. For each gaze data stream, the analyst could choose the AOI of the referee who was being observed and see if the referee covered his/her primary AOI. Afterward, with a press of a button, save all entries could be saved into a csv file, followed by the next frame appearing automatically for all four videos (Figure 2). In the end, this csv file could be exported and used for further analyses.

### Case Study

#### Participants and Design

A single-case study with a team of expert referees was chosen for this study. This team (all males) adjudicated a preparation game of the German Basketball Bundesliga (Ref A, age = 29, experience on German expert level = 9; Ref B, age = 36, experience = 10; Ref C, age = 44, experience = 17; all with experience at the international level). All of them had normal or corrected-to-normal vision.

#### Ethics Statement

All participants provided written informed consent prior to their participation, in accordance to the principles outlined by the World Medical Association’s Declaration of Helsinki and the Office of Research Ethics at the German Sport University Cologne (ethics proposal number: 141/2018). Participants were initially told that the study would investigate their communication strategy after a call during a game. After the game, they were informed about the real aim of the study.

#### Apparatus

Three Pupil Core mobile eye-tracking systems (Pupil Labs GmbH, Berlin, Germany) were used in this study. We used the binocular mobile eye-tracking headset connected to the mobile bundle which composed of a Motorola Moto Z2 or Z3 Play with an USBC-USBC cable. The eye movements were recorded with 200 Hz and were matched to the simultaneously captured scene videos recorded at 30 Hz (30 frames per second).

#### Procedure

Prior to testing, the referees were informed about the procedure. It was explained to them how eye tracking devices work, and they were given instructions on how to handle them. One hour before the game, the eye tracking headsets were adjusted to the participants. Thereafter, the referees conducted their normal pre-game warm-up without the eye tracking systems until 5 min before the start of the game. Then, the referees and examiners met in front of the scorer’s table. The eye tracking systems were returned to the referees and the *Manual Marker Calibration* was conducted (Kassner et al., 2014). The referees then placed themselves on the free-throw line and looked toward the basket. One of the examiners held a Pupil Calibration Marker v0.4 in his/her hand and placed himself/herself 1 m in front of the referees. The referees were told not to move their heads and to follow the route of the marker only with their eyes. A predefined route was used to calibrate every single eye-tracker. At half-time, the recordings were stopped and 5 min before the second half, the same calibration procedure was repeated. Shortly before these calibration processes, the eye trackers were synchronized by time *via* a visual signal. The recordings were saved on an SD-card and calibrated *post hoc* using the Pupil Player application (version 1.17, Pupil Labs GmbH, Berlin, Germany; Kassner et al., 2014).

#### Data

To test the new developed analysis tool, this case study was conducted in line with a game specific task–in this case, *the behaviors of the referees in critical foul situations according the guidelines*. An expert–a 52-years-old basketball professional with experience as a player, a coach and a referee on national level–reviewed the whole game and rated 23 scenes as highly challenging foul situations with critical decisions. The scenes were shortlisted based on the choice of the expert on relevant time periods before the call/decision. For every frame, the experimenter determined whether the referees’ gaze was inside the primary observation area (according to FIBA guidebook) or not. Furthermore, for every frame, the coordinated gaze behavior was assessed on the basis of whether the referees looked at the same AOI or not. The AOI were defined as the ball, the basket, another referee, the scorer’s table, defending or attacking players, the zone, and the three-point line.

### Data Analysis by Using the New Tool

#### Data Integration and Synchronization

After launching the analysis tool, the control (base) window and four other smaller windows (called view0, view1, view2, and view3) are displayed on the screen. After saving a new project, a window appears where the path name of the video files needs to be inserted. In addition to the selection of the video file, the analyst has to insert the videos’ frame rate in ms (e.g., 40 ms = 25 Hz; 33.33 ms = 30 Hz). He/she can also select whether the audio track of the video file is played or not and the way the windows (view0–3) are arranged. The program also allows the synchronization of videos.

#### Steps of Analysis

**FIGURE 3:**
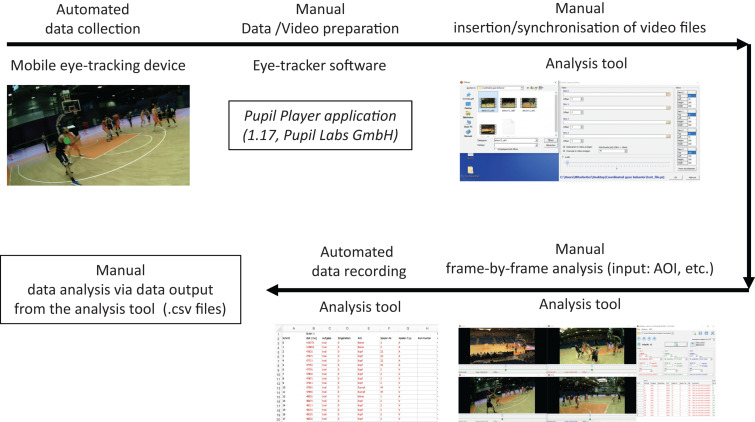
Flow chart of the working process for analyzing gaze coordination in dynamic events using the new tool.

After clicking on the *analysis button*, the program will start with the first frame. For the recordings of the gazes (of the referees), the appropriate AOI (ball, basket, scores table or shot clock) or player (attacking or defending, including the jersey number) can be chosen. In our study, we could also record if the referees covered the primary area of responsibility. Moreover, an additional insert field to add comments for each column is available. After the analyst completes entries for one frame, all the relevant information is saved automatically followed by access to the next frame. In the end, a csv file is available with all the data and can be exported and used for further analyses. Figure 3 represents a flowchart of the working process, highlighting how the simultaneous analysis of multiple persons’ gaze behavior is possible using the new tool in dynamic events.

## Results and Discussion

The primary aim of this study was to develop and apply, for the first time, a method to investigate the simultaneous gaze behavior of a team of three people operating in a highly dynamic environment. One major challenge in such an investigation is the large amount of data generated. Our method provides a solution for this problem by presenting a procedure which allows easy classification of large datasets and subsequently, makes analyses easier.

The method we have developed is not only economical, but also offers a potential for wide applicability. Using mobile eye-tracking devices in highly dynamic and specialized situations could affect the natural behavior of the participants. However, in our study, the participants did not report any sizeable constraints during the process of data collection despite the game activity. They all reported that they needed a short period of time to familiarize themselves with the eye-tracking device and after a few minutes, they didn’t feel any constraints.

The recording of the gaze behaviors of the participants during the chosen scenes within the basketball game, did not present any major problems either and the gaze points of all the participants were clearly visible. The use of the analysis tool makes frame-by-frame analysis of gaze recordings of multiple participants feasible, because all the three videos (of the referees) could be analyzed simultaneously, and no additional computer program was needed for manually transferring the data from the recorded videos. Previous studies have presented approaches to analyze simultaneous gazes of dyads in laboratory studies (e.g., Neider et al., 2010) or the simultaneous gazes of even bigger groups in a digital classroom (Nyström et al., 2017). The highly technical demands of investigating gaze of a group has been the focus of these investigations and past results have shown that such paradigms work well with acceptable deviations in different technical parameters (e.g., latency, Nyström et al., 2017). Based on these results, Scurr et al. (2014) have stated that several studies show methodologies for simultaneous gaze analysis, but none of them has been applied in highly dynamic situations. As an innovative extension of these investigations and research methods, the methodological approach presented in this study, allows an analysis of teams’ gaze behavior in dynamic actions. Even beyond that, it can be applied to other research fields, such as social cognition in which the presence of another person has been found to affect the gaze of an observer (e.g., Nasiopoulos et al., 2015); or this approach could help to assess how social attention is distributed in multi-agent contexts (cf. Dalmaso et al., 2020).

*Results of the case study:* The analysis included 2,339 video frames synchronized per referee. The mean duration of the scenes lasted 106.31 frames (*SD* = 40.36). In 47.07% of the analyzed scenes, the referees looked at a point in their primary observation area (with a high *SD* = 24.35%). This distribution of the coverage of the primary observation area varied as a function of the referees’ positions (trail 54.58%, *SD* = 21.30; lead 39.03%, *SD* = 24.85; center 47.67, *SD* = 26.14). Analyzing the coordination of the referees’ gaze, in just 5.61% of all analyzed frames, all three referees fixated their gaze on the same area. In 31.94% of all analyzed frames, two referees fixated their gaze on the same area (center + lead 13.34%; center + trail 10.90%; lead + trail 7.70%). The results showed that the referee team under observation followed the FIBA guidelines in approximately half of all included cases (playing situations). Interestingly, in majority of the analyzed frames (>90%), they distributed their gaze to different AOI to cover as many aspects of game actions as possible. In contrast, the single case study of Fasold et al. (2018) showed that the novice referees in handball did not coordinate their gaze behavior in an appropriate manner, i.e., both referees focused on areas close to the ball in about 80% of the analyzed data. Considering that in our study the tested team of referees was highly experienced, it does seem surprising that deviations from the guidelines occur here as well.

## Conclusion

The method of analyzing synchronized gaze behavior of three individuals seems promising for future efforts in various other scenarios involving many individuals who try to work together as a team (e.g., personal protection, traffic monitoring). Furthermore, it could be used to replicate findings of basic experimental research on gaze behavior of teams (e.g., Bahrami et al., 2010; Neider et al., 2010) in natural environments or settings.

We were able to show that similar simultaneous gaze evaluation with more than one or two participants is possible in dynamic situations, enabling new possibilities in studying social and functional coordinated interactions in future. We extended the approach by Fasold et al. (2018) to three referees working together as a team, with the aim of perceiving as much of the relevant game actions as possible in a very information-rich environment. This study shows that using mobile eye-tracking devices (Pupil Labs) and a new analysis tool does make simultaneous gaze analyses in dynamic environments possible and even more efficient. To develop the analysis tool we utilized commonly used components (e.g., PubLibVlc) because these components guarantee high performance, stability and compatibility with all available video formats. The manual frame-by-frame analysis is still time consuming but, it can be conducted much faster considering the availability of software that facilitates the analytical process. This manual method may be necessary until machine-learning processes allow algorithmic-based data analysis in highly dynamic scenarios. In our case, we expect that the manual analysis will allow the report of frequencies next as a qualitative observation of the recorded data (game actions) and this could result in a step toward automated analysis. Although, the tool we developed is available on GIThub, we recommend adjusting the program to the specific needs of every researcher’s own project. Furthermore, we suggest advancements to the new analysis tool by integrating keyboard short cuts or the development of a specialized keyboard like the ones used for video editing. These adaptations could further optimize the user interface of the analysis tool and could optimize manual data analysis.

To conclude, the current status of our software should be seen as a starting point for investigations into coordination of visual skills in groups, not only in sports, but also in everyday tasks where acting as a team is required to achieve the defined objectives.

## Data Availability Statement

The original contributions presented in the study are included in the article/supplementary material, further inquiries can be directed to the corresponding author/s.

## Author Contributions

FF, KS, BN, and SK developed the study concept and contributed to the design. AN collected the data and was supported by FF, KS, BN, and SK. AN analyzed the data together with FF, FS, KS, BN, and SK. FS developed the analysis tool. FF and SK wrote the first draft of the manuscript. All authors helped to edit and revise the manuscript and approved the final submitted version of the manuscript.

## Conflict of Interest

The authors declare that the research was conducted in the absence of any commercial or financial relationships that could be construed as a potential conflict of interest.

## Footnotes

1 https://prog.olsztyn.pl/paslibvlc/

2 https://www.tmssoftware.com

3 https://github.com/Seifriz/AFVS
